# Supplementary material for: The Systems Biology Research Tool: evolvable open-source software
Source: BMC Syst Biol. 2008 Jun 29;2:55. doi: 10.1186/1752-0509-2-55 (PMC2446383; doi:10.1186/1752-0509-2-55)
Supplement: Additional file 1 — SBRT Archive. An archive of the current version of the Systems Biology Research Tool. [file 1752-0509-2-55-S1.zip › sbrt-1.4.0/doc/developers_guide/api/sbrt/kernel/math/DoubleEquiv.html]

DoubleEquiv


|  |  |  |
| --- | --- | --- |
| **Overview** \| **Package** \| **Class** \| **Use** \| **Tree** \| **Deprecated** \| **Index** \| **Help** | |  |
| **PREV CLASS**   **NEXT CLASS** | **FRAMES**    **NO FRAMES**     **All Classes** |  |
| SUMMARY: NESTED \| FIELD \| CONSTR \| METHOD | DETAIL: FIELD \| CONSTR \| METHOD |  |


---


## sbrt.kernel.math Class DoubleEquiv

```
java.lang.Object
  sbrt.kernel.math.DoubleEquiv
```

**All Implemented Interfaces:**: EquivTester<java.lang.Double>

---

``` public class DoubleEquiv extends java.lang.Object implements EquivTester<java.lang.Double> ```

This class is used to compare double precision numbers for equivalence.

**Author:**
:   This class was written and documented by Jeremiah Wright while in the
    Wagner lab.

---

**Constructor Summary**

`DoubleEquiv(double tolerance)`
  
          Creates a new double precision number equivalence tester with the
provided tolerance.


| **Method Summary** | |
| --- | --- |
| `boolean` | `areEquiv(double value1, double value2)`             Indicates if the provided values are equivalent. |
| `boolean` | `areEquiv(java.lang.Double value1, java.lang.Double value2)`             Indicates if the provided values are equivalent. |
| `double` | `getTolerance()`             Returns the tolerance used by this equivalence tester. |

**Methods inherited from class java.lang.Object**

`clone, equals, finalize, getClass, hashCode, notify, notifyAll, toString, wait, wait, wait`

**Constructor Detail**

### DoubleEquiv

```
public DoubleEquiv(double tolerance)
```

:   Creates a new double precision number equivalence tester with the
    provided tolerance.

    **Parameters:**: `tolerance` - the maximum amount by which two doubles can differ, but still be considered equivalent.


**Method Detail**

### getTolerance

```
public double getTolerance()
```

:   Returns the tolerance used by this equivalence tester.

    :   **Returns:**: the maximum amount by which two doubles can differ, but still be considered equivalent.

---


### areEquiv

```
public boolean areEquiv(java.lang.Double value1,
                        java.lang.Double value2)
```

:   Indicates if the provided values are equivalent.

    :   **Specified by:**: `areEquiv` in interface `EquivTester<java.lang.Double>`
    :   **Parameters:**: `value1` - a value.: `value2` - a value. **Returns:**: `true` if the provided values are equivalent, `false` otherwise.

---


### areEquiv

```
public boolean areEquiv(double value1,
                        double value2)
```

:   Indicates if the provided values are equivalent.

    :   **Parameters:**: `value1` - a value.: `value2` - a value. **Returns:**: `true` if the provided values are equivalent, `false` otherwise.


---


|  |  |  |
| --- | --- | --- |
| **Overview** \| **Package** \| **Class** \| **Use** \| **Tree** \| **Deprecated** \| **Index** \| **Help** | |  |
| **PREV CLASS**   **NEXT CLASS** | **FRAMES**    **NO FRAMES**     **All Classes** |  |
| SUMMARY: NESTED \| FIELD \| CONSTR \| METHOD | DETAIL: FIELD \| CONSTR \| METHOD |  |


---
